# Advancing prediction of age-related vascular cognitive impairment based on peripheral and retinal vascular health in a pilot study: a novel comprehensive assessment developed for a prospective workplace-based cohort (The Semmelweis Study)

**DOI:** 10.1007/s11357-024-01447-y

**Published:** 2024-11-27

**Authors:** Tamas Csipo, Agnes Lipecz, Peter Mukli, Anna Péterfi, Zsofia Szarvas, Anna Ungvari, Lamyae El Alaoui, Márton Sándor, Attila Kállai, Mónika Fekete, Gábor Á. Fülöp, Stefano Tarantini, Anna Csiszar, Zoltán Benyó, Péter Sótonyi, Adam G. Tabak, Béla Merkely, Andriy Yabluchanskiy, Zoltan Ungvari

**Affiliations:** 1 Institute of Preventive Medicine and Public Health, Faculty of Medicine, Semmelweis University, Budapest, Hungary; 2 Vascular Cognitive Impairment, Neurodegeneration and Healthy Brain Aging Program, Department of Neurosurgery, University of Oklahoma Health Sciences Center, Oklahoma City, OK USA; 3 Oklahoma Center for Geroscience and Healthy Brain Aging, University of Oklahoma Health Sciences Center, Oklahoma City, OK USA; 4 Stephenson Cancer Center, University of Oklahoma, Oklahoma City, OK USA; 5 Department of Health Promotion Sciences, College of Public Health, University of Oklahoma Health Sciences Center, Oklahoma City, OK USA; 6 International Training Program in Geroscience, Doctoral College, Health Sciences Program/Institute of Preventive Medicine and Public Health, Semmelweis University, Budapest, Hungary; 7 Department of Translational Medicine, Semmelweis University, Budapest, Hungary; 8 Eötvös Loránd Research Network and Semmelweis University (ELKH-SE) Cerebrovascular and Neurocognitive Disorders Research Group, Budapest, H-1052 Hungary; 9 Department of Vascular and Endovascular Surgery, Semmelweis University, Budapest, Hungary; 10 UCL Brain Sciences, University College London, London, UK; 11 Department of Internal Medicine and Oncology, Semmelweis University, Faculty of Medicine, Budapest, Hungary; 12 Heart and Vascular Center, Semmelweis University, Budapest, Hungary

**Keywords:** Healthy aging, Microcirculation, Vascular cognitive impairment and dementia

## Abstract

With a growing elderly population in the European Union, age-related diseases associated with unhealthy aging pose increasing public health challenges, including a loss of independence and heightened societal burdens. The Semmelweis Study, a prospective occupational cohort study in Hungary, seeks to identify determinants of unhealthy aging, focusing on the complex relationship between lifestyle, environmental, occupational factors, and the development of chronic age-associated diseases, including age-related vascular cognitive impairment (VCI). The primary objective of this pilot study was to establish a robust, high-throughput assessment methodology to comprehensively evaluate both peripheral and cerebrovascular health to provide a solid foundation for the forthcoming Semmelweis Study framework. The study involved 49 participants aged 23 to 87 years, and it assessed multi-domain cognitive performance through an automated battery of tests (CANTAB). Vascular health was comprehensively evaluated using laser speckle contrast imaging (LSCI), flow-mediated dilation (FMD), static and dynamic retinal vessel analysis (SVA, DVA), and measurements of vascular stiffness. The retinal microvasculature, which closely mirrors the cerebral circulation in anatomy, physiology, and pathophysiology, provided a unique window for examination. Optical imaging through SVA and DVA enables the identification of structural and functional changes in the central nervous system’s microcirculation, which are highly relevant to the pathogenesis of VCI. Subsequently, the collected measures were integrated into vascular health indices using principal component analysis (PCA) and the relationship to the age and cognitive status of study participants was explored. These comprehensive vascular health indices demonstrated a correlation not only with age but also with cognitive performance. This methodology holds promise for providing novel insights into the intricate interplay between vascular and cognitive health within the context of the Semmelweis Study.

**Supplementary Information:**

The online version contains supplementary material available at 10.1007/s11357-024-01447-y.

## Introduction

The aging of populations is a critical challenge faced by societies across the European Union (EU) and beyond [1]. The EU and the UK currently house over 100 million individuals aged 65 years and older, a number expected to surge to nearly 150 million by 2050 [1, 2]. This demographic shift, accounting for 21.2% in 2022 and projected to reach 29.5% of the population of the EU in 2050, underscores the pressing importance of addressing aging-associated diseases as a burgeoning public health concern [3]. Moreover, as the elderly population expands, the potential strain on societal resources due to age-associated diseases and loss of independence necessitates proactive measures for a sustainable future [4]. Safeguarding the health of aging populations is integral to achieving the Sustainable Development Goals (SDGs), with the realization that increased lifespan should be coupled with extended healthspan. This aspiration requires multifaceted efforts, spanning from disease prevention to health promotion, involving collaboration across sectors and engagement of the public. Importantly, significant health differences among EU member states magnify the challenge of adapting to these demographic shifts. Hungary, as an EU member, confronts the complexities of a rapidly aging and declining population. The proportion of individuals aged 65 or older in Hungary is projected to rise from 20.3% in 2021 to 27.9% by 2050 [5, 6]. The dynamic interplay between demographics, public health, labor markets, and social services presents Hungary with intricate challenges that necessitate a comprehensive understanding of aging-related health concerns and the determinants of successful/healthy aging.

In response to these imperatives, Semmelweis University (Budapest, Hungary) — Hungary’s largest healthcare provider, a major employer in the healthcare sector, and a leading health sciences institution in Central Europe — has launched the Semmelweis Caring University Model Program [7]. This multifaceted initiative is composed of three key elements: the Semmelweis Study [8], an extensive longitudinal epidemiological survey emphasizing healthy aging; the Semmelweis Workplace Health Promotion Model Program; and the Center for Preventive Services, a pioneering model program aimed at primary healthcare reform and the promotion of healthy aging.

The Semmelweis Study, a cornerstone of this initiative, is a prospective workplace cohort study, aiming to unravel the complex factors that contribute to unhealthy aging in Hungary [8]. It encompasses all employees and faculty members at Semmelweis University aged 25 years and older, a population of more than 12,000 people, capturing a diverse range of occupational and socioeconomic backgrounds. This comprehensive approach enables an in-depth examination of the complex network of factors including lifestyle choices, environmental influences, and occupational risks in in the workplace. The study focuses on how these diverse factors collectively determine healthspan and play a role in the development and progression of age-related chronic diseases.

One major aim of the Semmelweis Study is to investigate the complex relationship between vascular health and age-related cognitive impairment [9, 10]. Vascular cognitive impairment and dementia (VCID), comprising a range of cerebrovascular diseases, stand as prominent contributors to cognitive decline in older adults [11–13]. The study delves into the role of cerebral microcirculation, particularly its structural and functional changes with age, as a cornerstone of VCID’s pathogenesis. As risk factors such as hypertension, diabetes, atherosclerosis [9, 10], and smoking exacerbate the aging-related decline in cerebromicrovascular health, understanding these mechanisms becomes vital for the Semmelweis Study. One central goal is to pinpoint the determinants of cerebromicrovascular aging within Hungary’s aging population, illuminating potential avenues for interventions.

Embedded within the broader scope of the Semmelweis Study, an innovative workplace-based health promotion program has been introduced: the Semmelweis Workplace Health Promotion Model Program. This initiative is designed with the overarching objective of preserving and improving cardiovascular and cerebrovascular health. By integrating workplace-based strategies for health promotion, the Semmelweis Study not only seeks to deepen its understanding of age-related cognitive decline but also actively strives to enhance the health and well-being of the study’s participants and, by extension, the broader community. This innovative approach reflects a holistic commitment to advancing the science of aging while making a tangible impact on the lives of the workforce of Semmelweis University.

Within the intricate interplay between vascular health and cognitive function, cerebromicrovascular endothelial function and microvascular dilation have a pivotal role [14–16]. Clinical studies demonstrate age-related decline of microvascular function [17, 18] and murine models highlight a causal link between deterioration of microvascular dilation and cognitive dysfunction during aging [19, 20] and in pathological conditions characterized by accelerated microvascular aging. Remarkably, interventions designed to counter the effects of aging exhibit considerable promise in rejuvenating cerebromicrovascular endothelial function, leading to associated cognitive improvements [21–23]. The Semmelweis Study, along with its integrated intervention programs, keenly attuned to these interconnections, strives to clarify the intricate web linking vascular health and cognitive performance.

Within the scope of the Semmelweis Study, an important objective emerges: the development of a robust, high-throughput assessment methodology to quantify the health status of both peripheral and brain vasculature. This approach extends to establishing clear correlations between vascular health metrics and cognitive function, thus facilitating the early prediction and prevention of age-related cognitive decline. In pursuit of these goals, the study embraces an array of cutting-edge approaches, including assessment of micro- and macrovascular peripheral endothelial health using laser speckle contrast imaging (LSCI) and flow-mediated dilation (FMD) approaches, measures of vascular stiffness, and static and dynamic vessel analysis (SVA and DVA) as proxies for the structural and functional health of brain microvasculature. The integration of these metrics into a comprehensive index offers a panoramic view of vascular health, unveiling potential links to cognitive function. The primary objective of the present study is to perform preliminary investigations and refine methodologies, thereby laying the solid groundwork for the forthcoming comprehensive assessment of vascular health within the broader Semmelweis Study framework.

## Methods

### Recruitment and study population

The study was advertised through flyers at primary care clinics of the participating institutions. Exclusion criteria were the following: acute cardiovascular disease within 6 months of visit, presence of peripheral artery disease, active cancer, chemotherapy within 12 months of visit, renal disease, hepatic disease, acute infectious disease, untreated hypertension, untreated diabetes, major neuropsychiatric disorder (diagnosis of dementia, schizophrenia, etc.). Educational status, smoking status, and habits (such as alcohol consumption) were noted, and all prescribed medications were recorded. The presence of lipid disorders and thyreopathies were determined based on the prescribed medications. All procedures in the present study were approved by the Hungarian Medical Research Council (53,981–2/2023/809) and the Institutional Review Board of the University of Oklahoma Health Sciences Center (8129, 9384, 9555). Eligible participants were enrolled after they were familiarized with the complete protocol and all participants provided informed consent prior to inclusion.

### Measurement of peripheral macrovascular endothelial function using ultrasonography-based flow-mediation dilation (FMD) testing

FMD was performed according to the American College of Cardiology guidelines as previously published [24]. Blood pressure was measured in a seated position; participants were then asked to rest in a supine position for 10 min. Brachial artery diameter was imaged using an Acuson Sequoia 256 (Acuson, Mountain View, CA, USA) ultrasound device equipped with an 8-MHz linear sonography probe (Acuson 8L5). To induce FMD, a rapid cuff inflator system (Hokanson E20 Rapid Cuff Inflator, Hokanson, Bellevue, WA, USA) was utilized, with a cuff positioned below the antecubital fossa of the right arm. The cuff pressure was maintained at 50 mmHg above systolic blood pressure for a duration of 5 min. Following cuff occlusion release, vascular diameter measurements were recorded for a period of 3 min. The FMD was calculated as a percentage change from the maximum achieved vessel diameter relative to the baseline diameter. The acquired images underwent semiautomatic processing utilizing the Brachial Analyzer for Research software (Medical Imaging Applications LLC, Coralville, IA, USA). Any negative changes in diameter or FMDs exceeding 15% were excluded from the subsequent analysis.

### Measurement of peripheral microvascular endothelial function using laser speckle contrast imaging (LSCI)-based approach

**Fig. 1:**
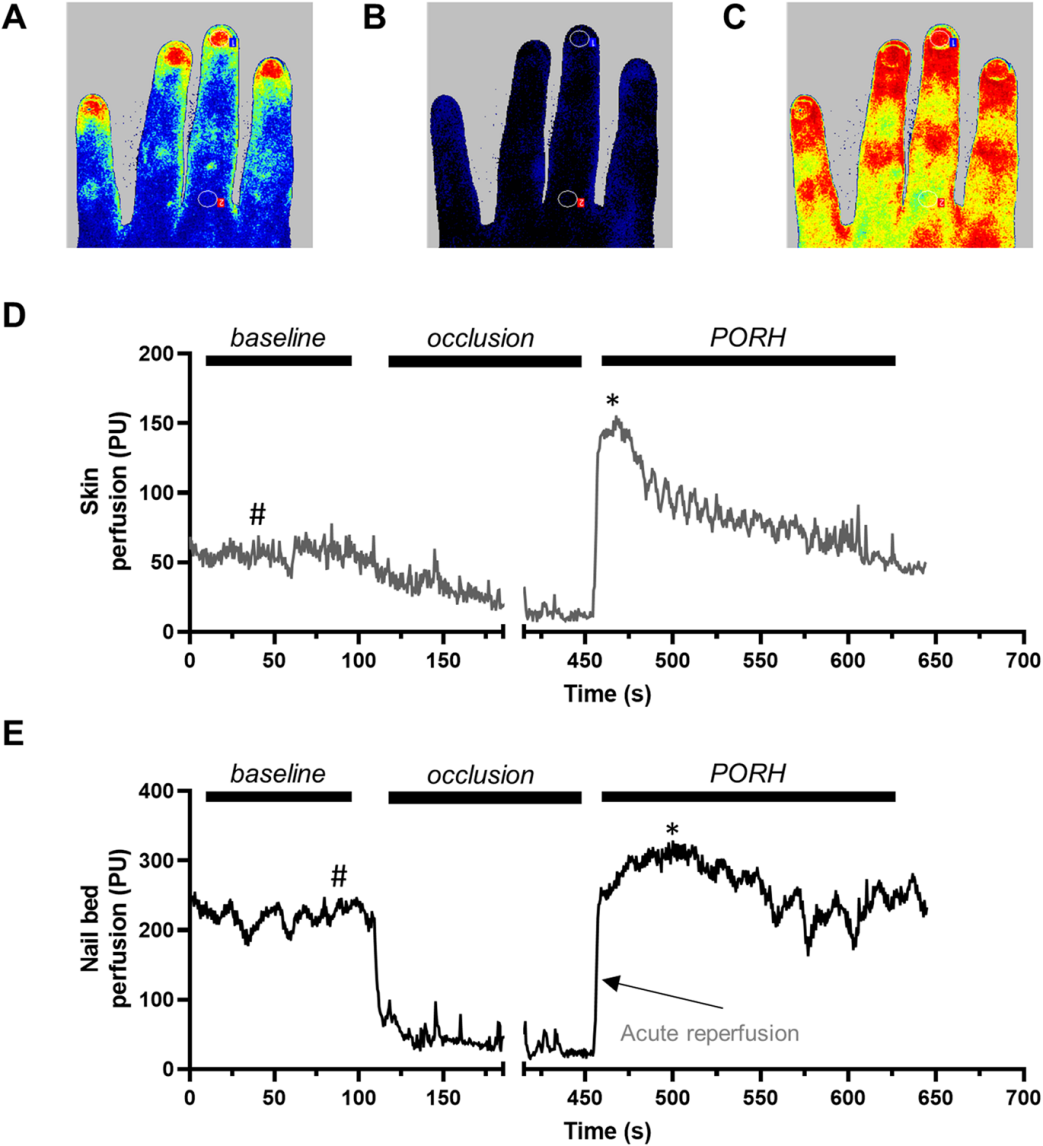
Representative peripheral microvascular health assessment of a 33-year-old male using laser speckle contrast imaging (LSCI). Peripheral microvascular health was assessed using LSCI during a post-occlusive reactive hyperemia (PORH) test on the back of the left hand of each participant. After stabilization of baseline perfusion, measurements were taken for both the skin and nail bed areas (indicated by the pound symbol, “#,” on **D** and **E**). To reduce blood perfusion in the arm to the biological minimum, a blood pressure cuff on the upper arm was inflated to 50 mmHg above systolic blood pressure for 5 min. After cuff occlusion was released, post-occlusive changes in tissue perfusion were monitored, and maximal perfusion rates were recorded (indicated by the asterisk symbol, “*,” on **D** and **E**). Acute reperfusion was assessed as the slope of reperfusion in the first 4 s after cuff occlusion was released (**E**). **A** Pseudocolor perfusion image obtained during baseline conditions. **B** Pseudocolor perfusion image obtained during the occlusion phase. **C** Pseudocolor perfusion image obtained during PORH. In **A–C**, warmer colors represent higher perfusion rates, while colder colors represent lower perfusion rates. **D** Perfusion recording during a complete PORH testing procedure obtained over the skin of the 1st phalanx of the middle finger. **E** Perfusion recording during a complete PORH testing procedure obtained over the skin of the nail bed of the middle finger

FMD testing was followed by assessment of microvascular reactivity using LSCI during a post-occlusive reactive hyperemia (PORH) test [24, 25]. LSCI imaging was performed using a Perimed PSI-NR system (Perimed, Järfälla, Sweden) positioned approximately 20 cm above the left hand of seated patients. After 5 min of resting in a seated position, baseline skin perfusion was recorded for at least 60 s after it has stabilized. A sphygmomanometer cuff was then inflated above the antecubital fossa to a pressure of 50 mmHg above systolic blood pressure for 5 min, similar to the protocol used to occlude peripheral blood flow during FMD testing. After release of cuff occlusion, changes in skin perfusion were recorded for at least 2 min. Skin temperature was noted after measurement at the first and last phalanxes of the middle finger with a non-contact thermometer (Thermoworks TW2, Thermoworks, American Fork, UT, USA). Baseline and maximal post-occlusive perfusion values of the two regions, the skin of the back of the middle finger (Fig. 1D), and the nail bed (Fig. 1E) were evaluated offline. Maximal perfusion values relative to baseline perfusion values were used for further analysis (Fig. 1). Reperfusion velocity, the parameter describing the acute reperfusion phase of PORH, was defined by the slope of the initial 4 s of reperfusion after the release of cuff occlusion (Fig. 1).

### Assessment of vascular stiffness

Arterial stiffness was assessed by pulse waveform analysis (PWA). Pressure waveform signals were recorded using SphygmoCor CVMS (AtCor Medical, Naperville, IL, USA) [26] in the radial artery after patients rested in a supine position for 10 min. Arterial stiffness was evaluated by the calculation of aortic augmentation indices. Recordings with operator indices (a measure of recording quality) < 90% were excluded from further analysis.

### The use of static and dynamic retinal vessel analysis (SVA and DVA) as a proxy measure of cerebrovascular health

**Fig. 2:**
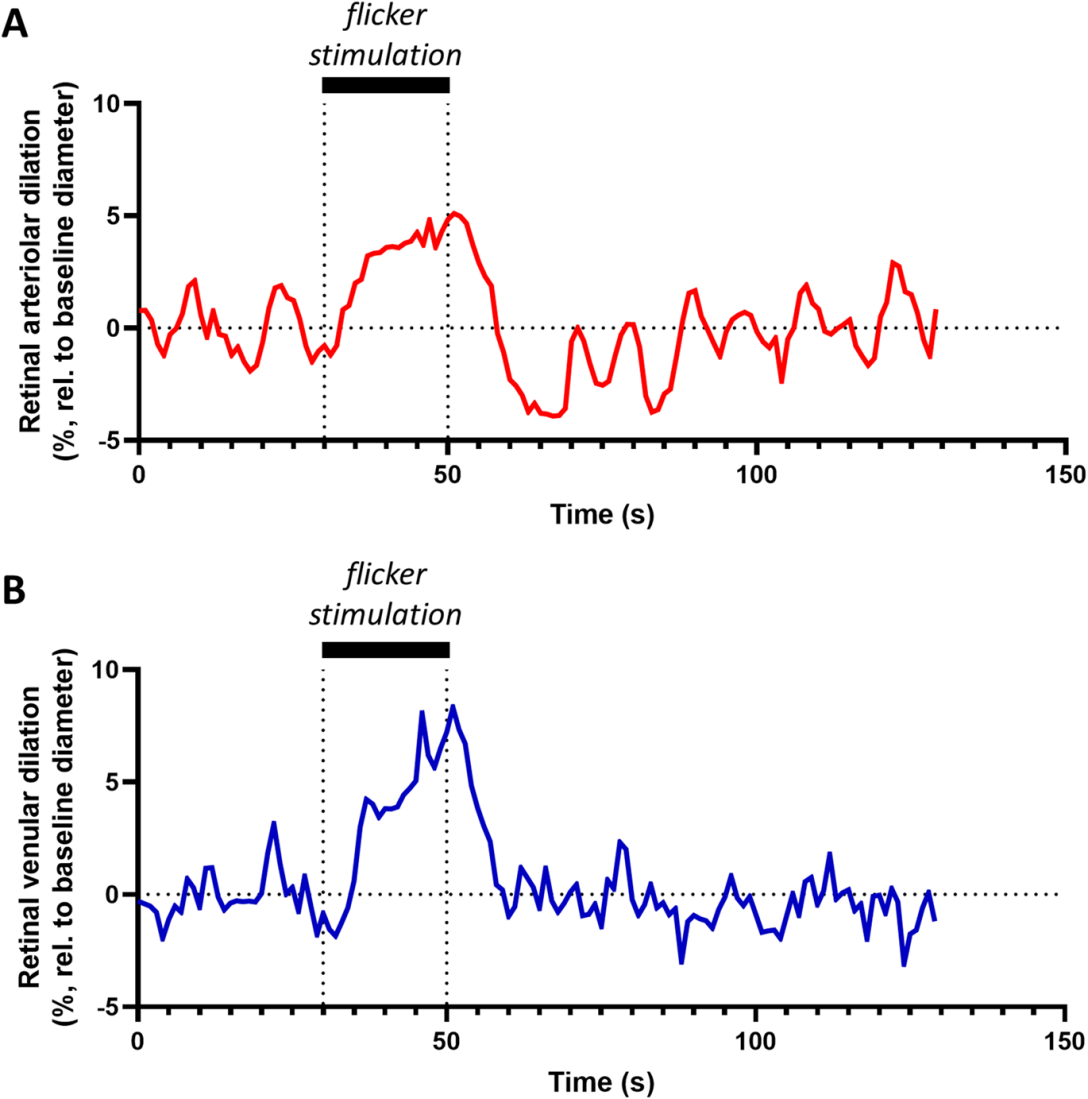
Representative retinal microvascular health assessment of a 29-year-old female using dynamic retinal vessel analysis (DVA). Microvascular health in the central nervous system was assessed in the retina using DVA. Following pupil dilation, patients were seated at a mydriatic fundus camera and were instructed to maintain fixation on a designated target. Retinal arteriolar (**A**) and venular (**B**) diameters were monitored contiuously before, during, and after flicker light stimulus. Maximal change in vessel diameter relative the baseline was obtained during this examination

Retinal vessel assessments were performed as previously described [27]. To provide a succinct overview, we initiated pupil dilation in the right eye using 1% topical tropicamide (USP, AKORN, Lake Forest, IL, USA). Patients were instructed to maintain fixation on a designated target, ensuring that the area of interest was centered within the fundus image. Measurement of retinal arteriolar and venular diameters was carried out utilizing the dynamic vessel analyzer (DVA) developed by IMEDOS Systems in Jena, Germany, in accordance with established protocols [28, 29]. The DVA device facilitated the noninvasive and continuous assessment of retinal vessel diameters along a designated vessel segment, both before and after flicker light stimulation. Vessel segments of 1-mm length were evaluated for mean maximal arteriolar dilation and mean maximal venular dilation in response to flicker light stimulation [30]. The flicker light employed maintained a frequency of 12.5 Hz and matched the wavelength of the illuminating light. Duration of flicker stimuli was 20 s. Prior to initiating flicker stimulation, we recorded a baseline measurement for a minimum duration of approximately 100 s (Fig. 2). To ensure accuracy, we computed the statistical mean from three consecutive examinations for each subject and each assessed parameter.

Static retinal vessel assessment (SVA) of arterio-venous ratio was calculated from central retinal arteriolar (CRAE) and central retinal venular (CRVE) equivalents using the Paar-Hubbard formula [31].

### Cognitive testing

Cognitive testing was performed via a computerized cognitive test that consisted of several tasks of the Cambridge Automated Neuropsychological Test Automated Battery (CANTAB, Cambridge Cognition, Cambridge, UK). Tests that are capable of detecting fine aging-associated changes were selected based on previously published data [32]. A motor screening task was completed to make sure participants were capable of using a touchscreen device, as tests were performed on a 10.5-in. iOS (Apple, Cupertino, CA, USA) tablet. Memory was assessed via the delayed matching to sample (DMS), and paired associates learning (PAL) tests. Attention and psychomotor speed were tested via the reaction time (RTI) and rapid visual information (RVP) tests. Executive function and working memory were tested via the spatial working memory (SWM) test.

### Statistical analysis

Plotting of representative figures and univariate statistical tests were performed in GraphPad Prism 9.x (GraphPad Software, La Jolla, CA, USA). Normal distribution of data was tested with d’Agostino-Pearson normality test. On normally distributed data, Student’s *t*-test was used for group comparisons, and mean ± SD are reported. On non-normally distributed data, Mann–Whitney *U*-test was performed, and medians with interquartile ranges (IQR) are reported. All other statistical tests and calculations were performed in SPSS (IBM Corp., Armonk, NY, USA). To derive composite scores of cognitive function and vascular health, data dimension was reduced using principal component analysis (PCA) with varimax rotation. Prior to PCA, correlation analysis of variables within each PCA was performed and any variable with a correlation coefficient of 0.85 or higher was removed. The adequacy of the sample was assessed using the Kaiser–Meyer–Olkin (KMO) index and the Bartlett sphericity test. The KMO values > 0.50 and *p* < 0.05 for the Bartlett test were considered acceptable. Three PCA-based score extractions were performed, and the component scores were used as composite indices describing cognitive impairment, peripheral vascular health, and peripheral vascular health augmented with retinal vascular health in each participant. The Cognitive Impairment Index (CII) consisted of parameters yielded by the automated cognitive test. CII input variables consisted of the number of errors or latency measured during each task; therefore, a higher score value represented worse cognitive performance. Another PCA-based dimension reduction was performed on peripheral vascular parameters (FMD, max/baseline perfusion measured over the skin, max/baseline perfusion measured over the nail bed, reperfusion velocity, and the augmentation index), which yielded the vascular health indices (VHI) of participants. The third PCA-based extraction (prVHI = peripheral and retinal Vascular Health Index) consisted of the variables in VHI, and additionally, retinal arteriolar and venular change in diameter evoked by flicker light together with the arteriovenous ratio was included. Scores for extracted components with an eigenvalue > 1 in each PCA were used for further analysis. To test the association of cognitive and vascular assessment-derived variables, Pearson correlation analysis was used, and in case of the correlation of raw retinal vascular parameters and raw cognitive performance parameters, an additional age-controlled analysis was performed.

## Results

### Participants

A total of 49 participants of 23–87 years of age were enrolled in current study. Population characteristics are summarized in Table 1 and Table 2. A total of 30 complete FMD measurements were included in the analysis. Measurements with visible motion artefacts or implausible values (not between 0 and 15%) were excluded from further analysis. Complete microcirculatory assessments were available for 41 participants, and measurements were excluded from further analysis if motion artifacts were present within seconds of the peak (maximal) perfusion during the PORH phase. Complete PWA measurements were available for all 49 participants. SVA and arterio-venous ratio assessments were completed for 47 participants: two were excluded due to the semi-automated software not recognizing several vessels. DVA parameters were available for all participants. In terms of cognitive assessments, complete data were available for 45 participants, with only domain-specific results being excluded for four participants. During the cognitive RVP test, one young participant and two older participants deviated from the task. One older participant did not complete the cognitive DMS test. It is worth noting that no participant reported regular alcohol consumption.

**Table 1:** Population characteristics: age, blood pressure, heart rate, and endothelial function

	*Mean*	*SD*	*Unit*
Age	50.5	20.3	Years
*Males*	50.7	21.8	
*Females*	48.8	19.6	
Systolic blood pressure	117.7	13.1	mmHg
Diastolic blood pressure	73.4	8.3	mmHg
Heart rate	64.1	8.5	1/min
Body mass index (BMI)	24.8	3.8	kg/m^2^
Flow-mediated dilation (FMD)	6.7	4.3	%

**Table 2:** Population characteristics: sex, education, comorbidities, and prescribed medication

	*n*	***%***
Sex		
*Male*	23	46.9
*Female*	26	53.1
Education		
*High school graduate*	1	2
*Bachelor’s degree*	15	3.6
*Master’s degree*	10	20.4
*Professional doctorate*	8	16.3
*Academic doctorate*	11	22.5
*Other*	4	8.2
Hypertension	13	26.5
Diabetes	2	4.1
Hyperlipidemia	9	18.4
Active smoker	1	2
Anti-hypertensive use		
*ACEI*	3	6.1
*ARB*	3	6.1
*Alpha*_*1*_*-blocker*	1	2
*Beta-blocker*	2	4.1
*CCB*	1	2
*ACEI* + *diuretic*	1	2
*ACEI* + *CCB*	1	2
*ARB* + *diuretic*	1	2
Anti-diabetic use		
*DPP-4 inhibitor*	1	2
*DPP-4 inhibitor* + *biguanide*	1	2
Hormone replacement		
*Thyroid*	6	12.2
*Estrogen*	3	6.1

*ACEI* angiotensin converting enzyme inhibitor, *ARB* angiotensin receptor blocker, *CCB* Ca^2+^-channel blocker, *DPP-4 inhibitor* dipeptidyl peptidase-4 inhibitor

### Retinal vascular markers predict cognitive performance in multiple domains

To investigate the potential convergence of age-related cognitive decline and age-associated alterations in retinal vascular function as a proxy measure of cerebrovascular health, we conducted a comprehensive correlation analysis between cognitive performance markers and parameters related to retinal vascular function. Additionally, we performed a separate analysis in which correlations were adjusted for the participants’ age. The outcomes of these correlation analyses are succinctly summarized in Table 3.

**Table 3:** Retinal vascular markers predicting cognitive performance in several domains

		Retinal arteriolar dilation (%)		Retinal venular dilation (%)	Arterio-venous ratio
-none-^a^	Age (years)	*r*	− 0.181	− 0.334*	− 0.460**
		*p*	*0.265*	***0.035***	***0.003***
	DMSMLAD	*r*	− 0.310	− 0.293	− 0.289
		*p*	*0.051*	*0.066*	*0.071*
	DMSPC	*r*	− 0.154	0.056	0.342*
		*p*	*0.342*	*0.732*	***0.031***
	PALFAMS28	*r*	− 0.038	0.205	0.489**
		*p*	*0.815*	*0.206*	***0.001***
	PALTEA28	*r*	0.054	− 0.191	− 0.476**
		*p*	*0.742*	*0.239*	***0.002***
	RTIFMDMT	*r*	− 0.316*	− 0.132	0.076
		*p*	***0.047***	*0.417*	*0.640*
	RTIFMDRT	*r*	− 0.451**	− 0.386*	− 0.021
		*p*	***0.003***	***0.014***	*0.899*
	RVPA	*r*	0.037	**0.433**	0.100
		*p*	*0.819*	***0.005***	*0.539*
	RVPMDL	*r*	− 0.323*	− 0.367*	− 0.190
		*p*	***0.042***	***0.020***	*0.241*
	SWMBE4	*r*	0.040	− 0.055	− 0.210
		*p*	*0.809*	*0.735*	*0.194*
	SWMBE6	*r*	− 0.077	− 0.229	− 0.355*
		*p*	*0.638*	*0.154*	***0.024***
	SWMBE8	*r*	− 0.206	− 0.240	− 0.265
		*p*	*0.203*	*0.136*	*0.099*
	SWMS	*r*	− 0.087	− 0.176	− 0.190
		*p*	*0.593*	*0.278*	*0.240*
Age (years)	DMSMLAD	*r*	− 0.262	− 0.176	− 0.115
		*p*	*0.107*	*0.283*	*0.487*
	DMSPC	*r*	− 0.244	− 0.080	0.205
		*p*	*0.134*	*0.628*	*0.211*
	PALFAMS28	*r*	− 0.158	0.037	0.328*
		*p*	*0.335*	*0.823*	***0.041***
	PALTEA28	*r*	0.177	− 0.019	− 0.310
		*p*	*0.280*	*0.907*	*0.055*
	RTIFMDMT	*r*	− 0.265	0.051	0.414**
		*p*	*0.103*	*0.759*	***0.009***
	RTIFMDRT	*r*	− 0.433**	− 0.352*	0.069
		*p*	***0.006***	***0.028***	*0.674*
	RVPA	*r*	− 0.043	0.343*	− 0.114
		*p*	*0.796*	***0.033***	*0.488*
	RVPMDL	*r*	− 0.273	− 0.233	0.085
		*p*	*0.093*	*0.154*	*0.606*
	SWMBE4	*r*	0.117	0.078	− 0.048
		*p*	*0.480*	*0.636*	*0.773*
	SWMBE6	*r*	0.033	− 0.050	− 0.127
		*p*	*0.843*	*0.761*	*0.440*
	SWMBE8	*r*	− 0.123	− 0.051	0.018
		*p*	*0.455*	*0.757*	*0.914*
	SWMS	*r*	0.019	0.018	0.098
		*p*	*0.907*	*0.911*	*0.551*

Arteriovenous ratio and retinal venular dilation exhibited associations with chronological age. However, after adjusting for age, only parameters related to reaction time were correlated with retinal vascular dilation induced by flickering light. Furthermore, the arteriovenous ratio emerged as a predictor of visual memory, learning performance, and reaction time after age adjustment

*Abbreviations*: *DMSMLAD*, DMS mean correct latency (all delays); *DMSPC*, DMS percent correct; *PALFAMS28*, PAL first attempt memory score; *PALTEA28*, PAL total errors; *RTIFMDMT*, RTI median five-choice movement time; *RTIFMDRT*, RTI median five-choice reaction time; *RVPA*, RVPA’ (A prime), signal detection measure of subject sensitivity to target sequence; *RVPMDL*, RVP median response latency; *SWMBE4*, SWM between error (4 boxes); *SWMBE6*, SWM between error (6 boxes); *SWMBE8*, SWM Between error (8 boxes); *SWMS*, SWM Strategy

^*^p < 0.05, **p < 0.01

^a^Cells contain zero-order (Pearson) correlations

We observed significant correlations between retinal arteriolar dilation and reaction time parameters (RTIFMDMT, RTIFMDRT, and RVPMDL) prior to accounting for age. Additionally, retinal venular dilation displayed significant correlations with reaction time measures (RTIFMDMT and RVPMDL), as well as with the accuracy of the rapid visual information processing task (RVPA). Notably, the arteriovenous ratio of retinal vessels exhibited significant correlations with performance on visual memory tasks (DMSPC, PALFAMS28, PALTEA28), and the number of errors made during a spatial working memory task (SWMBE6).

Upon adjusting for the age of participants, the correlation between retinal arteriolar dilation and reaction time (RTIFMDRT) persisted. Moreover, significant correlations were found between retinal venular dilation and both reaction time (RTIFMDRT) and performance during the rapid visual information processing task (RVPA). Furthermore, arteriovenous ratio maintained its correlation with performance on a visual memory task (PALFAMS28) and the motion time during the reaction time test (RTIFMDMT).

### Exploration of association between cognitive impairment and vascular health indices

To evaluate the effectiveness of a comprehensive vascular assessment in predicting cognitive impairment, we employed principal component analysis (PCA) to reduce the dimension of data into a single score for each participant. This analysis resulted in the generation of three key indices: the Cognitive Impairment Index (CII), the Vascular Health Index (VHI), and the Peripheral + Retinal Vascular Health Index (prVHI). We subsequently conducted a thorough examination of the correlations between these PCA-generated indices. A summary of the correlation analysis results is presented in Table 4, while detailed descriptions of the individual components, including variable loadings and eigenvalues, can be found in Supplementary Tables 1–9. Notably, three components of the Cognitive Impairment Index (CII–c1, CII–c2, CII–c3) exhibited significant correlations with the age of the participants. This observation indicated a noteworthy association between aging and cognitive function, with positive correlations suggesting aging-associated cognitive impairment. Furthermore, the second component of the Vascular Health Index (VHI–c2) and the second and third components of the Peripheral + Retinal Vascular Health Index (prVHI–c2 and pfVHI–c3) also displayed significant correlations with age. In this context, negative correlations between age and vascular health indices suggested aging-associated deterioration of vascular function. Additionally, positive correlations were identified between the third component of the Peripheral + Retinal Vascular Health Index (prVHI–c3) and the fourth component of the Cognitive Impairment Index (CII–c4). Conversely, negative correlations were observed between the first component of VHI (VHI–c1) and the first component of CII (CII–c1), the second component of prVHI (prVHI–c2) and the third and fourth components of CII (CII–c3 and CII–c4), and the third component of prVHI (prVHI–c3) and the second component of CII (CII–c1). These negative correlations indicated a significant association between deteriorating vascular function and impaired cognitive performance.

**Table 4:** Correlation between compoments generated by principal component analysis describing cognitive impairment and vascular health (peripheral and peripheral + retinal)

		Peripheral vascular health (VHI–c1)	Peripheral vascular health (VHI–c2)	Peripheral + retinal vascular health (prVHI c1)	Peripheral + retinal vascular health (prVHI c2)	Peripheral + retinal vascular health (prVHI c3)	Age
Cognitive performance (CII – c1)	*r*	− 0.453*	− 0.188	− 0.365	− 0.091	− 0.287	0.420**
	*p*	***0.023***	*0.369*	*0.095*	*0.688*	*0.196*	***0.005***
Cognitive performance (CII – c2)	*r*	− 0.074	− 0.335	− 0.001	− 0.143	− 0.425*	0.405**
	*p*	*0.726*	*0.101*	*0.996*	*0.526*	***0.049***	***0.006***
Cognitive performance (CII – c3)	*r*	− 0.033	− 0.249	0.043	− 0.453*	− 0.139	0.544**
	*p*	*0.877*	*0.230*	*0.850*	***0.034***	*0.536*	***0.000***
Cognitive performance (CII – c4)	*r*	− 0.051	0.204	− 0.020	− 0.480*	0.424*	− 0.121
	*p*	*0.807*	*0.328*	*0.929*	***0.024***	***0.049***	*0.435*
Age	*r*	− 0.376	− 0.590**	− 0.206	− 0.483*	− 0.580**	
	*p*	*0.079*	***0.001***	*0.235*	***0.008***	***0.001***	

The components within the cognitive impairment and vascular health indices exhibit significant associations with chronological age. Additionally, the components within the vascular health indices demonstrate significant correlations with components describing cognitive performance

^*^p < 0.05, **p < 0.01

## Discussion

In this pilot study, we have successfully demonstrated the association between cognitive performance and the function and health of small vessels in the central nervous system, as assessed through retinal examination. Notably, some of the correlations between retinal vascular health measures and cognitive performance weakened when we accounted for the age of participants, indicating that both cognitive changes and vascular alterations are closely linked to the aging process.

We used a principal component analysis (PCA)-based method to characterize vascular health with a composite score (VHI) [24]. The first component of VHI significantly correlated with the first component of the cognitive impairment scores (CII), reaffirming previous findings [24]. These two indices encapsulated changes in both peripheral circulation and cognitive function, explaining most of the variability within our aging cohort. While the first component of VHI (VHI – c1) did not exhibit a significant correlation with age, the second component of VHI (VHI–c2) did show a significant negative correlation with age, indicating that aging-associated factors contributed to the variance in these peripheral and retinal vascular parameters. It is noteworthy that the vascular health indices that significantly correlated with the age of participants contained peripheral vascular endothelial function (FMD) with a higher loading value. Peripheral endothelial function measured via FMD declines after a certain age [33], and lower FMD is also associated with a decline in cognitive performance [34]. The acute peripheral reperfusion in the microcirculation represented by reperfusion velocity during LSCI was incorporated with lower or negative loading values in the components that did not correlate with age, whereas this phase of hyperemia may also be influenced by the age of individuals [24]. When observing the loadings of the prVHI components, AVR together with retinal arteriolar dilation (prVHI2) or retinal venular dilation (prVHI3) were represented with higher loading values. The correlations between VHI, prVHI, and CII components were not significant in all cases, which may be caused by the different loading values for the cognitive performance metrics in each CII. The association of certain vascular health parameters and cognitive performance metrics proved to be cognitive domain specific, as evidenced by the association of individual cognitive performance and retinal vascular health metrics shown in Table 3.

By incorporating retinal parameters into the vascular health scores (prVHI), we established a more robust assessment methodology capable of quantifying the overall health status of the vasculature in both the periphery and the central nervous system. This integrated vascular health metric, derived from various standard and novel techniques including LSCI, DVA, FMD, and vascular stiffness measurements, provided comprehensive insights. The first component of prVHI (prVHI – c1) did not significantly correlate with age, aligning with the VHI based on peripheral vascular metrics. However, both the second and third prVHI components (prVHI – c2 and prVHI – c3) exhibited significant correlations with age, highlighting that aging-associated factors significantly contributed to the variance in these peripheral and retinal vascular parameters. Importantly, the second component of prVHI (prVHI – c2) correlated with CII –c3, indicating that major age-related changes in cognitive function were associated with the deterioration of peripheral and retinal vascular health.

Our approach enables us to explore the correlations between vascular health status and cognitive function within the Semmelweis Study cohort, providing valuable insights into the determinants of vascular health and facilitating the early prediction and prevention of age-related cognitive decline. Retinal vascular assessments emerge as a standout tool within our methodology. Static and dynamic retinal vascular analyses yielded vascular parameters capable of predicting cognitive performance across different domains. Given the shared embryologic origin of the retina and brain tissue, retinal vessels closely mirror the structural and physiological alterations of brain microvessels. The sensitivity of DVA to age-related changes in retinal neurovascular coupling underscores its potential in detecting cerebromicrovascular and neurovascular dysfunction, thereby enriching the comprehensiveness of our assessment.

In clinical studies, the assessment of CBF regulation in the cerebral circulation is rarely paired with the assessment of peripheral vascular parameters. To bridge this gap, we combined retinal assessments (both static and dynamic) with the gold standard methodology for assessing endothelial function in human studies, namely the ultrasonography-based FMD test [35, 36]. Age-related changes in endothelial function can be detected by FMD [37] which is sensitive to the presence of systemic cardiovascular risk factors [36, 38, 39], highlighting its significance in our comprehensive assessment. LSCI also plays a pivotal role in our methodology, offering the ability to assess peripheral microcirculatory dysfunction. In contrast to FMD, LSCI provides valuable insights into endothelial function in smaller vessels and the microcirculation [41]. By monitoring changes in blood perfusion within the superficial skin and skin appendages, LSCI allows detection of aging-associated microvascular changes, thus enriching our understanding of systemic vascular health [24, 42]. Age-related arterial stiffening, along with macro- and microvascular dysfunction, is a well-documented phenomenon [45, 46]. However, these macro- and microvascular changes rarely occur in isolation, underscoring the importance of a comprehensive assessment that encompasses both peripheral and central nervous system circulation. Such an approach holds promise in predicting functional changes within the central nervous system.

When considering potential alternative methods for obtaining information about the health status of blood vessels in the brain, it is crucial to acknowledge the opportunities and challenges they present. Techniques such as magnetic resonance imaging (MRI) and functional magnetic resonance imaging (fMRI) offer valuable insights into cerebral vascular integrity and function. Nonetheless, physical and economic constraints limit the routine use of MRI and fMRI, especially in large-scale prospective cohort studies [47]. Transcranial Doppler sonography (TCD) is a lightweight, more economical method that can measure age-related changes in cerebral blood flow dynamics related to neurovascular dysfunction [18, 47, 48]. However, TCD monitoring may not be successful in a large fraction of participants due to anatomic variations in the skull, which can obstruct insonation of large arteries at the base of the brain. While these methods offer unique advantages, they may be better suited for embedded studies within the Semmelweis Study, where they can validate our approach and delve into specific biological questions in greater depth.

To generate integrated indices, we employed PCA, using results from various vascular measurements to create comprehensive parameters. This approach allowed us to condense complex data into single metrics, enhancing our ability to assess overall vascular health. Furthermore, we conducted statistical analyses to correct for the influence of aging, revealing that the relationship between vascular health and cognitive function remained significant even after accounting for age-related influences.

However, as with any study, there are limitations to consider. This pilot study involved a small number of participants, which may affect the generalizability of our findings. The number of participants limited the possibility to control for several factors that may influence cognitive function and vascular health. In the current pilot study, socioeconomic status, lifestyle factors and previous diseases were not assessed in depth. However, an inverse relationship is known between socioeconomic status and cardiovascular risk [49]. The risk of cardiovascular diseases is also determined by lifestyle factors, such as nutritional factors [50] or sleep [51]. Previous diseases (e.g., COVID-19) may also have a profound effect of both cognitive performance [52] and vascular health [53, 54]. In the Semmelweis Study, a wider outreach to potential study participants will results in higher observation numbers, and the refined methodology will also allow the investigation of the socioeconomic factors, lifestyle factors, or previous diseases on both cognitive and vascular function [8].

The current study is a cross-sectional study aiming to determine the feasibility of using the utilized methods in the larger cohort and determine whether a comprehensive vascular assessment can provide additional insight into the background of ageing-associated vascular cognitive impairment. Recruitment took place in a clinical/academic setting, which can also potentially influence the socioeconomic status and general health of participants. The Semmelweis Study cohort is also expected to be biased by the healthy worker effect and will not be representative of the general population [8]. Occupational cohort studies, however, have several strengths when compared to cohorts that are representative of a population. Higher participation rates lead to increased internal validity of results. Furthermore, based on another occupational cohort study, the Whitehall II study, the relative risks within the cohort have good external validity and are close to the risks observed in population-based trials [55].

FMD examinations are highly operator dependent and have suboptimal repeatability [40], which limit its ability to be used for repeated measures. In the current study, postocclusive reactive hyperemia was evoked with a paradigm similar to FMD during imaging with LSCI. Although specific limitations of LSCI use in longitudinal studies are currently unknown, the parameters recorded with the method offer reasonable to good repeatability [43]. Arterial stiffness was assessed through the measurement of augmentation indices that were derived from the pressure waveform captured over the radial artery. Aortic augmentation is a measure of systemic arterial stiffness [44], and is the contribution of the wave reflection coming from the periphery to the center to the systolic arterial pressure. Augmentation indices are calculated from the augmentation pressure, divided by the pulse pressure, and as both pressures increase with advanced age, a linear increase in this parameter with aging cannot be observed [56]. Alternatively, augmentation pressure or if methodology is available, pulse wave velocity should be also considered in large cohort studies as a measure of aging-associated arterial stiffening.

As SVA and DVA provide a unique opportunity for detecting microvascular changes in the central nervous system, the ocular vasculature may also be influenced by ocular specific diseases, such as glaucoma [57]. Hence, ophthalmologic disease screening should be performed on subjects to limit the effect of these confounders when using retinal vascular markers as surrogate markers for the cerebral microcirculatory function.

In the current study, PCA was used as a data reduction tool to generate composite scores for vascular health and the level of cognitive impairment. While PCA provides a valuable means of dimension reduction and summarizing complex datasets, it is important to acknowledge several limitations associated with its application in deriving indices, particularly in the context of our study on cognitive function and vascular health. PCA aims to capture the most significant sources of variance within the data. However, this approach may lead to a loss of specificity, as it condenses multiple variables into a smaller set of components. The components generated through PCA are linear combinations of the original variables, making them less straightforward to interpret compared to individual variables. Understanding the clinical significance of each component may require further investigation and validation. Additionally, PCA assumes a linear relationship between variables, which may not always hold true in complex biological systems. Non-linear relationships may be missed when using PCA, potentially leading to oversimplification of the underlying biological processes. In the process of dimension reduction, some amount of information is inevitably lost. In order to minimize the information lost, no variable was eliminated based on its correlation to the generated component, and all initially included variables were used for generation of the PCA-derived indices. To simplify the interpretation of data, correlation of the extracted components was not allowed in current analysis, as PCA with varimax rotation was used. This method assumes orthogonality (non-correlation) of components. In reality, correlation between variables describing cognitive performance or variables describing vascular health is possible. For example, correlation between cognitive performance metrics can be expected as prevailing theory suggests [58]. Therefore, alternative approaches, beyond PCA, may also hold promise for generating vascular health indices from the Semmelweis Study dataset. As the data collection progresses, the size of the dataset may allow the use of machine learning methods that handle non-linear relationships between variables and integrate multiple data types. Additionally, current methodology can be augmented with the measurement of biomarkers in biological samples (e.g., blood). Cardiovascular risk is associated to several known circulating factors (blood glucose, lipoproteins), and recent findings suggest that further information can be gained through analysis of biofluids with novel methods. Examination of circulating microvesicles serves as a form of liquid biopsy, which has emerged as a revolutionary strategy for diagnosis and prognosis prediction of vascular health [59] and cognitive impairment [60]. Application of these methods could offer different perspectives and insights, and future research may explore their application to enhance our understanding of the relationships between vascular health and cognitive function in the context of unhealthy aging.

In conclusion, in this pilot study, we have introduced a novel approach that incorporates peripheral and retinal vascular assessments within the Semmelweis Study. Our focus has been on the development of a robust assessment method and the establishment of meaningful correlations. This approach sets the stage for further investigations into the intricate connections between vascular health and age-related cognitive dysfunction. Looking ahead, as the Semmelweis Study expands its participant base, numerous possibilities emerge. These include the development of new indices and risk calculators, exploration of trajectories for cognitive and vascular health decline, and the potential for innovative research directions using our methodologies. The approach presented here not only enhances our understanding of cognitive decline but also offers a promising framework for early detection and intervention strategies in the realm of age-related cognitive dysfunction.

## Supplementary Information

Below is the link to the electronic supplementary material.

Supplementary file1 (DOCX 40 KB)

## Data Availability

The depersonalized data that support the findings of this study are available from the corresponding author upon reasonable request.
