# Supplementary material for: Advancing prediction of age-related vascular cognitive impairment based on peripheral and retinal vascular health in a pilot study: a novel comprehensive assessment developed for a prospective workplace-based cohort (The Semmelweis Study)
Source: GeroScience. 2024 Nov 27;47(1):1329–44. doi: 10.1007/s11357-024-01447-y (PMC11872852; doi:10.1007/s11357-024-01447-y)
Supplement: Supplementary file 1 — Supplementary file1 (DOCX 40 KB) [file 11357_2024_1447_MOESM1_ESM.docx]

**SUPPMENTAL MATERIAL**

**Advancing prediction of age-related vascular cognitive impairment based on peripheral and retinal vascular health: a novel comprehensive assessment developed for a prospective workplace-based cohort (The Semmelweis Study)**

Tamas Csipo^1,2,3, #^, Agnes Lipecz^1,2,3,#^, Peter Mukli^1,2,3,#^, Anna Péterfi^1,2,3^, Zsofia Szarvas^1,2,3^, Anna Ungvari^1^, Lamyae El Alaoui^1^, Márton Sándor^1^, Attila Kállai^1^, Mónika Fekete^1^, Gábor Á. Fülöp^2,3,12^, Stefano Tarantini^2,3,4,5,6^, Anna Csiszar^2,3,4,5,6^, Zoltán Benyó^7,8^, Péter Sótonyi^9^, Adam G. Tabak^1,10,11^, Béla Merkely^12^, Andriy Yabluchanskiy^2,3,4,5,6^  Zoltan Ungvari^2,3,4,5,6^

1) Institute of Preventive Medicine and Public Health, Faculty of Medicine, Semmelweis University, Budapest, Hungary

2) Vascular Cognitive Impairment, Neurodegeneration and Healthy Brain Aging Program, Department of Neurosurgery, University of Oklahoma Health Sciences Center, Oklahoma City, OK, USA

3) Oklahoma Center for Geroscience and Healthy Brain Aging, University of Oklahoma Health Sciences Center, Oklahoma City, OK, USA

4) Stephenson Cancer Center, University of Oklahoma, Oklahoma City, OK, USA

5) Department of Health Promotion Sciences, College of Public Health, University of Oklahoma Health Sciences Center, Oklahoma City, OK, USA

6) International Training Program in Geroscience, Doctoral College, Health Sciences Program/ Institute of Preventive Medicine and Public Health, Semmelweis University, Budapest, Hungary

7) Department of Translational Medicine, Semmelweis University, Budapest, Hungary

8) Eötvös Loránd Research Network and Semmelweis University (ELKH-SE) Cerebrovascular and Neurocognitive Disorders Research Group, Budapest H-1052, Hungary

9) Department of Vascular and Endovascular Surgery, Semmelweis University, Budapest, Hungary

10) UCL Brain Sciences, University College London, London, U.K.

11) Department of Internal Medicine and Oncology, Semmelweis University, Faculty of Medicine, Budapest, Hungary.

12) Heart and Vascular Center, Semmelweis University, Budapest, Hungary

**^#^**these authors contributed equally

Corresponding author**:**

Tamas Csipo, MD

Institute of Preventive Medicine and Public Health, Semmelweis University, Budapest, Hungary

Email: [csipo.tamas@semmelweis.hu](mailto:csipo.tamas@semmelweis.hu)

Running title: Vascular health assessment

**Supplementary Tables**

**Supplementary Table 1.**

An automated, tablet based cognitive testing method (CANTAB, Cambridge Cognition) was used to measure cognitive performance in the studied population. Major outcome variables (shown in Supplementary Table 2) were reduced into single cognitive impairment scores (Cognitive Impairment Index, CII) using principal component analysis with varimax rotation. The table contains the parameters that describe the variance explained by each extracted component.

| Component | Initial Eigenvalues | | | Extraction Sums of Squared Loadings | | | Rotation Sums of Squared Loadings | | |
| --- | --- | --- | --- | --- | --- | --- | --- | --- | --- |
|  | Total | % of Variance | Cumulative % | Total | % of Variance | Cumulative % | Total | % of Variance | Cumulative % |
| 1 | 4.551 | 37.927 | 37.927 | 4.551 | 37.927 | 37.927 | 3.041 | 25.343 | 25.343 |
| 2 | 2.036 | 16.965 | 54.891 | 2.036 | 16.965 | 54.891 | 2.315 | 19.291 | 44.634 |
| 3 | 1.201 | 10.011 | 64.902 | 1.201 | 10.011 | 64.902 | 2.145 | 17.878 | 62.512 |
| 4 | 1.028 | 8.566 | 73.468 | 1.028 | 8.566 | 73.468 | 1.315 | 10.956 | 73.468 |
| 5 | 0.834 | 6.951 | 80.419 |  |  |  |  |  |  |
| 6 | 0.618 | 5.146 | 85.566 |  |  |  |  |  |  |
| 7 | 0.467 | 3.890 | 89.456 |  |  |  |  |  |  |
| 8 | 0.436 | 3.632 | 93.088 |  |  |  |  |  |  |
| 9 | 0.353 | 2.939 | 96.026 |  |  |  |  |  |  |
| 10 | 0.230 | 1.915 | 97.941 |  |  |  |  |  |  |
| 11 | 0.149 | 1.240 | 99.181 |  |  |  |  |  |  |
| 12 | 0.098 | 0.819 | 100.000 |  |  |  |  |  |  |
| Extraction Method: Principal Component Analysis. | | | | | | | | | |

**Supplementary Table 2.**

An automated, tablet based cognitive testing method (CANTAB, Cambridge Cognition) was used to measure cognitive performance in the studied population. Major outcome variables (shown in the table) were reduced into single cognitive impairment scores (Cognitive Impairment Index, CII) using principal component analysis with varimax rotation. The table describes the loading of each variable to each extracted component prior to rotation.

**Abbreviations:**

DMSMLAD: DMS Mean Correct Latency (all delays); DMSPC: DMS Percent Correct; PALFAMS28: PAL First Attempt Memory Score; PALTEA28: PAL Total Errors; RTIFMDMT: RTI Median Five-Choice Movement Time; RTIFMDRT: RTI Median Five Choice Reaction Time; RVPA: RVPA’ (A prime), signal detection measure of subject sensitivity to target sequence; RVPMDL: RVP Median Response Latency; SWMBE4: SWM Between error (4 boxes); SWMBE6: SWM Between error (6 boxes); SWMBE8: SWM Between error (8 boxes); SWMS: SWM Strategy

|  | CII-c1 | CII-c2 | CII-c3 | CII-c4 |
| --- | --- | --- | --- | --- |
| DMSMLAD | 0.397 | 0.477 | 0.090 | 0.041 |
| DMSPC | -0.372 | 0.123 | 0.776 | -0.296 |
| PALFAMS28 | -0.799 | 0.047 | 0.201 | 0.411 |
| PALTEA28 | 0.740 | -0.056 | -0.362 | -0.444 |
| RTIFMDMT | 0.258 | 0.748 | 0.000 | 0.262 |
| RTIFMDRT | -0.004 | 0.717 | 0.165 | -0.447 |
| RVPA | -0.624 | -0.152 | 0.191 | 0.129 |
| RVPMDL | 0.455 | 0.611 | -0.143 | 0.448 |
| SWMBE4 | 0.653 | -0.500 | 0.108 | 0.212 |
| SWMBE6 | 0.811 | -0.245 | 0.288 | 0.201 |
| SWMBE8 | 0.816 | -0.036 | 0.314 | 0.028 |
| SWMS | 0.811 | -0.090 | 0.378 | 0.016 |
| Extraction Method: Principal Component Analysis. | | | | |

**Supplementary Table 3.**

An automated, tablet based cognitive testing method (CANTAB, Cambridge Cognition) was used to measure cognitive performance in the studied population. Major outcome variables (shown in the table) were reduced into single cognitive impairment scores (Cognitive Impairment Index, CII) using principal component analysis with varimax rotation. The table describes the loading of each variable to each extracted component after rotation.

**Abbreviations:**

DMSMLAD: DMS Mean Correct Latency (all delays); DMSPC: DMS Percent Correct; PALFAMS28: PAL First Attempt Memory Score; PALTEA28: PAL Total Errors; RTIFMDMT: RTI Median Five-Choice Movement Time; RTIFMDRT: RTI Median Five Choice Reaction Time; RVPA: RVPA’ (A prime), signal detection measure of subject sensitivity to target sequence; RVPMDL: RVP Median Response Latency; SWMBE4: SWM Between error (4 boxes); SWMBE6: SWM Between error (6 boxes); SWMBE8: SWM Between error (8 boxes); SWMS: SWM Strategy

|  | CII-c1 | CII-c2 | CII-c3 | CII-c4 |
| --- | --- | --- | --- | --- |
| DMSMLAD | 0.177 | 0.189 | 0.551 | 0.155 |
| DMSPC | 0.034 | -0.382 | -0.166 | 0.817 |
| PALFAMS28 | -0.383 | -0.835 | -0.068 | 0.023 |
| PALTEA28 | 0.248 | 0.897 | 0.035 | -0.111 |
| RTIFMDMT | -0.014 | 0.017 | 0.829 | 0.077 |
| RTIFMDRT | -0.278 | 0.246 | 0.434 | 0.643 |
| RVPA | -0.261 | -0.551 | -0.292 | 0.089 |
| RVPMDL | 0.146 | 0.072 | 0.855 | -0.212 |
| SWMBE4 | 0.759 | 0.176 | -0.142 | -0.324 |
| SWMBE6 | 0.878 | 0.206 | 0.117 | -0.122 |
| SWMBE8 | 0.778 | 0.320 | 0.230 | 0.075 |
| SWMS | 0.825 | 0.292 | 0.174 | 0.108 |
| Extraction Method: Principal Component Analysis. | | | | |
| Rotation converged in 7 iterations. | | | | |

**Supplementary Table 4.**

Multiple peripheral vascular assessments were performed on the studied population, including measurement of endothelial function via flow-mediated dilation (FMD) testing, measurement of peripheral microcirculatory reactivity with laser speckle contrast imaging (LSCI) and measurement of arterial stiffness via pulse waveform analysis (PWA). Major outcome variables (shown in Supplementary Table 4) were reduced into single vascular health scores (Vascular Health Index, VHI) using principal component analysis with varimax rotation. The table contains the parameters that describe the variance explained by each extracted component.

| Component | Initial Eigenvalues | | | Extraction Sums of Squared Loadings | | | Rotation Sums of Squared Loadings | | |
| --- | --- | --- | --- | --- | --- | --- | --- | --- | --- |
|  | Total | % of Variance | Cumulative % | Total | % of Variance | Cumulative % | Total | % of Variance | Cumulative % |
| 1 | 2.224 | 44.480 | 44.480 | 2.224 | 44.480 | 44.480 | 2.201 | 44.028 | 44.028 |
| 2 | 1.126 | 22.521 | 67.001 | 1.126 | 22.521 | 67.001 | 1.149 | 22.974 | 67.001 |
| 3 | 0.829 | 16.572 | 83.573 |  |  |  |  |  |  |
| 4 | 0.522 | 10.449 | 94.022 |  |  |  |  |  |  |
| 5 | 0.299 | 5.978 | 100.000 |  |  |  |  |  |  |
| Extraction Method: Principal Component Analysis. | | | | | | | | | |

**Supplementary Table 5.**

Multiple peripheral vascular assessments were performed on the studied population, including measurement of endothelial function via flow-mediated dilation (FMD) testing, measurement of peripheral microcirculatory reactivity with laser speckle contrast imaging (LSCI) and measurement of arterial stiffness via pulse waveform analysis (PWA). Major outcome variables (shown in Supplementary Table 4) were reduced into single vascular health scores (Vascular Health Index, VHI) using principal component analysis with varimax rotation. The table contains the parameters that describe the loading of each variable to each extracted component prior to rotation.

|  | VHI-c1 | VHI-c2 | |
| --- | --- | --- | --- |
| FMD (%) | 0.051 | 0.846 | |
| Skin max./baseline perfusion ratio | 0.763 | 0.340 | |
| Nail bed max./baseline perfusion ratio | 0.876 | -0.036 | |
| Reperfusion velocity (PU/s) | -0.569 | 0.540 | |
| Augmentation index (at HR=75/min) | 0.741 | 0.048 | |
| Extraction Method: Principal Component Analysis. | | |  |

**Supplementary Table 6.**

Multiple peripheral vascular assessments were performed on the studied population, including measurement of endothelial function via flow-mediated dilation (FMD) testing, measurement of peripheral microcirculatory reactivity with laser speckle contrast imaging (LSCI) and measurement of arterial stiffness via pulse waveform analysis (PWA). Major outcome variables (shown in Supplementary Table 4) were reduced into single vascular health scores (Vascular Health Index, VHI) using principal component analysis with varimax rotation. The table contains the parameters that describe the loading of each variable to each extracted component after rotation.

|  | VHI-c1 | VHI-c2 | |  |
| --- | --- | --- | --- | --- |
| FMD (%) | 0.172 | 0.830 | |  |
| Skin max./baseline perfusion ratio | 0.804 | 0.227 | |  |
| Nail bed max./baseline perfusion ratio | 0.861 | -0.161 | |  |
| Reperfusion velocity (PU/s) | -0.486 | 0.616 | |  |
| Augmentation index (at HR=75/min) | 0.740 | -0.059 | |  |
| Extraction Method: Principal Component Analysis. Rotation converged in 3 iterations. | | |  |  |

**Supplementary Table 7.**

Multiple vascular assessments were performed on the studied population, including measurement of endothelial function via flow-mediated dilation (FMD) testing, measurement of peripheral microcirculatory reactivity with laser speckle contrast imaging (LSCI), measurement of arterial stiffness via pulse waveform analysis (PWA), static and dynamic retinal vessel analysis (SVA and DVA). Major outcome variables (shown in Supplementary Table 5) were reduced into single peripheral and retinal vascular health scores (Peripheral + Retinal Vascular Health Index, prVHI) using principal component analysis with varimax rotation. The table contains the parameters that describe the variance explained by each extracted component.

| Component | Initial Eigenvalues | | | Extraction Sums of Squared Loadings | | | Rotation Sums of Squared Loadings | | |
| --- | --- | --- | --- | --- | --- | --- | --- | --- | --- |
|  | Total | % of Variance | Cumulative % | Total | % of Variance | Cumulative % | Total | % of Variance | Cumulative % |
| 1 | 2.953 | 36.908 | 36.908 | 2.953 | 36.908 | 36.908 | 2.591 | 32.381 | 32.381 |
| 2 | 1.615 | 20.184 | 57.092 | 1.615 | 20.184 | 57.092 | 1.670 | 20.878 | 53.260 |
| 3 | 1.120 | 14.005 | 71.097 | 1.120 | 14.005 | 71.097 | 1.427 | 17.837 | 71.097 |
| 4 | 0.717 | 8.959 | 80.056 |  |  |  |  |  |  |
| 5 | 0.696 | 8.705 | 88.761 |  |  |  |  |  |  |
| 6 | 0.402 | 5.025 | 93.786 |  |  |  |  |  |  |
| 7 | 0.280 | 3.497 | 97.283 |  |  |  |  |  |  |
| 8 | 0.217 | 2.717 | 100.000 |  |  |  |  |  |  |
| Extraction Method: Principal Component Analysis. | | | | | | | | | |

**Supplementary Table 8.**

Multiple vascular assessments were performed on the studied population, including measurement of endothelial function via flow-mediated dilation (FMD) testing, measurement of peripheral microcirculatory reactivity with laser speckle contrast imaging (LSCI), measurement of arterial stiffness via pulse waveform analysis (PWA), static and dynamic retinal vessel analysis (SVA and DVA). Major outcome variables (shown in Supplementary Table 5) were reduced into single peripheral and retinal vascular health scores (Peripheral + Retinal Vascular Health Index, prVHI) using principal component analysis with varimax rotation. The table contains the parameters that describes the loading of each variable to each extracted component prior to rotation.

|  | prVHI-c1 | prVHI-c2 | prVHI-c3 | |  |
| --- | --- | --- | --- | --- | --- |
| FMD (%) | 0.272 | 0.595 | 0.633 | |  |
| Skin max./baseline perfusion ratio | 0.824 | 0.012 | -0.093 | |  |
| Nail bed max./baseline perfusion ratio | 0.788 | -0.300 | -0.182 | |  |
| Reperfusion velocity (PU/s) | -0.345 | 0.701 | 0.060 | |  |
| Augmentation index (at HR=75/min) | 0.742 | -0.232 | 0.013 | |  |
| Arteriovenous ratio | 0.301 | 0.565 | -0.513 | |  |
| Retinal arteriolar dilation (%) | 0.658 | 0.547 | -0.238 | |  |
| Retinal venular dilation (%) | 0.621 | -0.082 | 0.595 | |  |
| Extraction Method: Principal Component Analysis. | | | |  |  |

**Supplementary Table 9.**

Multiple vascular assessments were performed on the studied population, including measurement of endothelial function via flow-mediated dilation (FMD) testing, measurement of peripheral microcirculatory reactivity with laser speckle contrast imaging (LSCI), measurement of arterial stiffness via pulse waveform analysis (PWA), static and dynamic retinal vessel analysis (SVA and DVA). Major outcome variables (shown in Supplementary Table 5) were reduced into single peripheral and retinal vascular health scores (Peripheral + Retinal Vascular Health Index, prVHI) using principal component analysis with varimax rotation. The table contains the parameters that describes the loading of each variable to each extracted component after rotation.

|  | prVHI-c1 | prVHI-c2 | prVHI-c3 | |  |
| --- | --- | --- | --- | --- | --- |
| FMD (%) | -0.114 | 0.186 | 0.884 | |  |
| Skin max./baseline perfusion ratio | 0.705 | 0.388 | 0.204 | |  |
| Nail bed max./baseline perfusion ratio | 0.840 | 0.197 | -0.023 | |  |
| Reperfusion velocity (PU/s) | -0.660 | 0.340 | 0.252 | |  |
| Augmentation index (at HR=75/min) | 0.753 | 0.118 | 0.154 | |  |
| Arteriovenous ratio | -0.002 | 0.817 | -0.067 | |  |
| Retinal arteriolar dilation (%) | 0.296 | 0.792 | 0.272 | |  |
| Retinal venular dilation (%) | 0.536 | -0.147 | 0.662 | |  |
| Extraction Method: Principal Component Analysis. Rotation converged after 5 interations. | | | |  |  |
